# Reply: Population change, population mixing and incidence of childhood acute lymphoblastic leukaemia in England and Wales

**DOI:** 10.1038/sj.bjc.6604553

**Published:** 2008-09-30

**Authors:** C A Stiller, M E Kroll, P J Boyle, Z Feng

**Affiliations:** 1 Childhood Cancer Research Group, Department of Paediatrics, University of Oxford, Oxford OX2 6HJ, UK; 2 School of Geography and Geosciences, Irvine Building, University of St Andrews, St Andrews KY16 9AL, UK

**Sir,**

We thank Professor Wakeford for his interest in our study of sociodemographic factors and childhood acute lymphoblastic leukaemia (ALL). We agree that it would be interesting to know whether incidence of ALL was related to population change since the previous census. Although 1981 population estimates for 1991 wards appear not to have been published, in the course of re-estimating census interaction data for 1981 according to the 1991 census geography for other purposes (Boyle and Feng, 2002), we calculated such estimates on the basis of a 1981 enumeration district (ED) to 1991 ward look-up table that had been constructed using a point-in-polygon method under a Geographical Information System platform. We were thus able to calculate the ratio of the 1991 to 1981 population estimates for all wards.

The population estimation method has some potential shortcomings, of which the most important is perhaps that 1981 ED centroids have only 100 m resolution; hence, in some cases, a 1981 ED near the boundary of a 1991 ward may have been allocated to the neighbouring ward. Ideally, the results should be validated by contacting local government planning agencies to check whether the wards with ratios at the extremes of the distribution were indeed those experiencing considerable decline or growth, but this was precluded by time and funding constraints.

We therefore felt it advisable to analyse only the data grouped into five categories defined by quintiles of the estimated population ratios. The minimum ratio was 0.47 and the maximum in the five quintile categories were 0.95, 1.00, 1.06, 1.13 and 83.3. Thus, approximately 40% of the wards experienced a population decrease between 1981 and 1991 and 60% experienced an increase. In general, however, the population changes were rather small, 5% or less of the 1981 population, in about 40% of wards. There was a highly significant positive trend linking quintile categories of 1991 : 1981 population ratio and diversity of origins of all-ages immigrants (*χ*^2^=215.6 on 1 df, *P*<0.0001). However, results of a univariate analysis of ALL incidence by quintile category of 1991 : 1981 population ratio are shown in Table 1, and there was no evidence that ALL risk was related to population ratio in the age groups 0 and 5–14 years. The tests for heterogeneity and trend were also non-significant for the age group 1–4 years; there was a suggestion of an increase, but the highest incidence was in the middle category of wards, those which experienced an increase of no more than 6% between the two censuses. These results may be only of fairly limited relevance to the question of whether the risk of childhood ALL is related to population increase, as population changes in most wards are unlikely to have taken place at a constant level throughout the period between the two censuses, and changes during 1991–1995 were ignored. A more sensitive test of the hypothesis might be possible if reliable estimates of ward populations could be obtained, perhaps from local government sources, for every year over a period of 10 years or more.

We are also grateful for the opportunity to correct an error in the penultimate paragraph of our article. As stated in the Results, in the multivariate analysis, there was a significant trend in incidence at ages 1–4 years in relation to the diversity of total incomers, and incidence was higher in rural wards than in those classified as urban or mixed. The interaction between these two variables was non-significant (*χ*^2^=10.1 on 8 df, *P*=0.26). When diversity of incomers was analysed separately for urban, mixed and rural wards, however, the heterogeneity and trend were only significant for urban wards (see Table 2). Incidence in rural wards in the highest quintile category for diversity of incomers was indeed higher than in any of the quintile categories of urban or mixed wards, but rural wards that were in the lowest two quintile categories for diversity of incomers had even higher rates.

## Figures and Tables

**Table 1:** Incidence rate ratios (IRRs) from univariate models for ALL by age group for wards classified according to quintiles of ratio of 1991 to 1981 census population estimates

	**Significance level**		**IRR in each quintile category**				
**Age group (years)**	**Heterogeneity**	**Trend**	**1 (decline)**	**2**	**3**	**4**	**5 (increase)**
0	0.62	0.40	1	1.42	1.10	1.18	1.44
1–4	0.12	0.08	1	1.00	1.19	1.08	1.11
5–14	1.00	0.99	1	0.97	0.99	0.98	1.00

**Table 2:** Incidence rates per million person-years and incidence rate ratios (IRRs) from univariate models for ALL diagnosed at ages 1–4 years for urban, mixed and rural wards classified according to quintiles of diversity of total incomers (1=lowest, 5=highest)

	**Significance level**		**Incidence in each quintile category**					**IRR in each quintile category**				
**Urban/rural status**	**Heterogeneity**	**Trend**	**1**	**2**	**3**	**4**	**5**	**1**	**2**	**3**	**4**	**5**
Urban	0.003	0.026	51.0	60.3	54.2	72.1	61.8	1	1.18	1.06	1.41	1.21
Mixed	0.72	0.31	53.7	67.5	65.9	62.7	71.5	1	1.26	1.23	1.17	1.33
Rural	0.44	0.13	89.0	88.6	60.0	61.3	76.3	1	1.00	0.67	0.69	0.86

Significance tests for heterogeneity and trend are within each level of urban/rural status. IRRs are presented with lowest quintile category of diversity of incomers as reference group.
